# Artificial intelligence–powered virtual standardized patients in teaching history-taking skills to medical students: a randomized controlled trial

**DOI:** 10.1186/s12909-026-09305-5

**Published:** 2026-04-30

**Authors:** Hai Nguyen Ngoc Dang, Thang Viet Luong, Hoa Thi Ha Vo, Linh Thi Khanh Nguyen, Trung Nguyen Tran, Hong Thi Anh Pham, Huong Thanh Truong, Hoa Tran, Toan Thanh Tran, Tien Anh Hoang, Thang Chi Doan, Quan Huynh, Thomas H. Marwick

**Affiliations:** 1 Faculty of Medicine, School of Medicine and Pharmacy, Duy Tan University, Da Nang, Vietnam; 2 Menzies Institute for Medical Research, University of Tasmania, Hobart, Tasmania Australia; 3 Nguyen Tat Thanh Hi-Tech Institute, Nguyen Tat Thanh University, Ho Chi Minh City, Vietnam; 4 Nguyen Tat Thanh University Center for Hi-Tech Development, Saigon Hi-Tech Park, Ho Chi Minh City, Vietnam; 5 199 Hospital, Da Nang, Vietnam; 6 Faculty of Medicine, Phenikaa University, Ha Noi, Vietnam; 7 Department of Internal Medicine, School of Medicine, University of Medicine and Pharmacy at Ho Chi Minh City, Ho Chi Minh City, Vietnam; 8 Cardiovascular Center, University Medical Center, Ho Chi Minh City, Vietnam; 9 Vietnam-Cuba Dong Hoi Hospital, Quang Tri, Vietnam; 10 Department of Internal Medicine, University of Medicine and Pharmacy, Hue University, Hue, Vietnam; 11 Hue Central Hospital, Hue, Vietnam; 12 Baker Heart and Diabetes Institute, Melbourne, Victoria Australia

**Keywords:** Artificial intelligence, Virtual standardized patient, History-taking skills, Medical education

## Abstract

**Background:**

Artificial intelligence (AI) has emerged as a promising tool in medical education. It offers opportunities to enhance learning experiences, particularly through the development of virtual standardized patients (SP) integrated with AI for training purposes. These virtual patients are especially useful for teaching history-taking skills (HTS). However, evidence comparing AI-powered virtual standardized patients (AI-VSP) with traditional SP in teaching HTS remains unclear. This study aimed to evaluate the effectiveness of AI-VSP and compare it with conventional SP in teaching HTS to undergraduate medical students.

**Methods:**

A randomized controlled trial (TCTR20251202012) was conducted among third-year medical students at Duy Tan University, Vietnam. This study included participants in the pre-clinical skills curriculum without prior formal HTS training. Students were randomized at the class level to intervention or control groups via a computer-generated sequence. The intervention group practiced with AI-VSP, while the control group learned with conventional SP. Primary outcomes included pre-test, post-test, and objective structured clinical examination (OSCE) scores. Examiners were blinded to group allocation. Student satisfaction was assessed as a secondary outcome using a 5-point Likert scale.

**Results:**

A total of 67 medical students were included, comprising 34 in the AI-VSP group and 33 in the SP group. Both groups demonstrated significant improvement in post-test scores compared with pre-test results (*p* < 0.001). In the AI-VSP group, the mean score increased from 3.41 ± 2.56 to 8.56 ± 1.85, while in the SP group, it rose from 3.24 ± 1.64 to 7.97 ± 1.85. The magnitude of improvement was 5.15 ± 2.88 in the AI-VSP group and 4.73 ± 1.75 in the SP group (*p* = 0.503). The mean OSCE scores were 6.5 ± 1.5 for the AI-VSP group and 6.3 ± 1.7 for the SP group (*p* = 0.543). Mixed-effects modeling adjusting for age and class-level clustering confirmed that neither pre-test, post-test, OSCE scores, nor score improvement differed significantly between instructional methods. Regarding student satisfaction, there was no statistically significant difference in perceived learning experience between the two groups (all *p* > 0.05).

**Conclusions:**

Learning outcomes and student satisfaction did not differ significantly between the AI-VSP and SP instructional methods.

**Trial registration:**

TCTR20251202012 (registered on 20/11/2025); retrospectively registered; https://www.thaiclinicaltrials.org/show/TCTR20251202012.

**Supplementary Information:**

The online version contains supplementary material available at 10.1186/s12909-026-09305-5.

## Background

History-taking skills (HTS) are among the fundamental competencies required for safe and effective medical practice [1]. Accordingly, HTS training is a compulsory component of undergraduate medical education. Teaching this skill through direct encounters with real patients allows students to bridge theoretical knowledge with clinical application. However, this traditional approach is often limited by safety, ethical, and legal concerns [2].

To address these limitations, the standardized patient (SP) method was developed as an alternative. SP provides students with immersive, clinically realistic experiences within a controlled environment. Numerous studies have demonstrated that SP-based teaching offers a consistent, reliable, and safe learning environment. It also effectively enhances the teaching of HTS. This method helps students gain confidence and procedural familiarity at simulation centers before transitioning to real clinical settings [3–6]. Despite its pedagogical strengths, this method demands substantial resources to achieve optimal results. Training SP requires significant time and manpower, and maintaining these programs incurs ongoing financial and logistical costs [7]. Over time, these challenges have prompted educators to explore more sustainable yet equally effective alternatives.

In recent years, artificial intelligence (AI) has emerged as a promising solution to support healthcare systems while reducing operational costs [8, 9]. Medical education, in particular, stands to gain significant benefits from AI innovations [10]. Evidence suggests that AI is playing an increasingly vital role at various educational levels, ranging from undergraduate medical training to residency and continuing medical education [11]. AI has been applied in diverse educational contexts, such as automated question generation [12], surgical skills training [13], and communication skill development [14].

Building upon these developments, the integration of AI-powered virtual standardized patients (AI-VSP) into medical education appears both feasible and beneficial. This approach could preserve the interactive and realistic features of SP-based instruction while addressing its major limitations related to cost, time, and human resources. In the context of teaching HTS, AI-VSP can provide students with standardized, repeatable, and responsive interactions that promote flexible practice and immediate feedback, thereby enhancing the learning experience.

Despite these potential advantages, there is still a lack of empirical evidence directly comparing conventional SP with AI-VSP in HTS education. Therefore, this study aimed to evaluate whether AI-VSP could achieve educational outcomes that differ from those of conventional SP. We hypothesized that AI-VSP would lead to potential improvements in students’ knowledge acquisition, objective structured clinical examination (OSCE) performance, and levels of satisfaction and engagement compared with traditional SP-based teaching.

## Methods

### Study design

This randomized controlled trial (Trial registration number: TCTR20251202012) was conducted at Duy Tan University in Da Nang, Vietnam. The study took place from April 2025 to October 2025. The study protocol was approved by Duy Tan University and fully adhered to the ethical principles of the Declaration of Helsinki (2013 version). Reporting of this trial followed the Consolidated Standards of Reporting Trials 2025 (CONSORT 2025) guidelines [15].

### Patient and public involvement

Patients and/or the public were not involved in the design, or conduct, or reporting, or dissemination plans of this research.

### Sample size and study participants

The study adopted a randomized controlled design. The intervention group was trained using AI-VSP, whereas the control group was taught with conventional SP. The primary outcome measure was the participants’ OSCE score. The study aimed to determine whether the AI-VSP method could lead to improved educational outcomes compared with the standard SP method, within a superiority trial framework [16].

The OSCE score was rated on a 10-point scale. Based on prior literature in medical education, a between-group difference of 1 point (10% of the total score) was considered the minimum educationally meaningful improvement [17]. A standard deviation (SD) of 0.65, converted from previously reported data on a 100-point scale, was used for the sample size estimation [18]. Assuming a two-sided significance level of 0.01 and a statistical power of 95%, the required sample size was calculated to be 15 participants per group. Allowing for a 10% expected attrition rate, the final target sample size was 17 students per group, resulting in a total of 34 participants [19].

Participants were third-year medical students enrolled in the pre-clinical skills curriculum at Duy Tan University. These students had not previously received formal training or practical experience in history-taking and voluntarily participated in the study. The pre-clinical course aimed to equip students with essential competencies, including presentation and reasoning skills, basic physical examination techniques, and familiarity with common clinical procedures and patient interactions. All participants had completed foundational medical science courses prior to the study. Students who were repeating the course were excluded. A total of 67 eligible students were recruited and enrolled in the study.

### Randomization

**Fig. 1:**
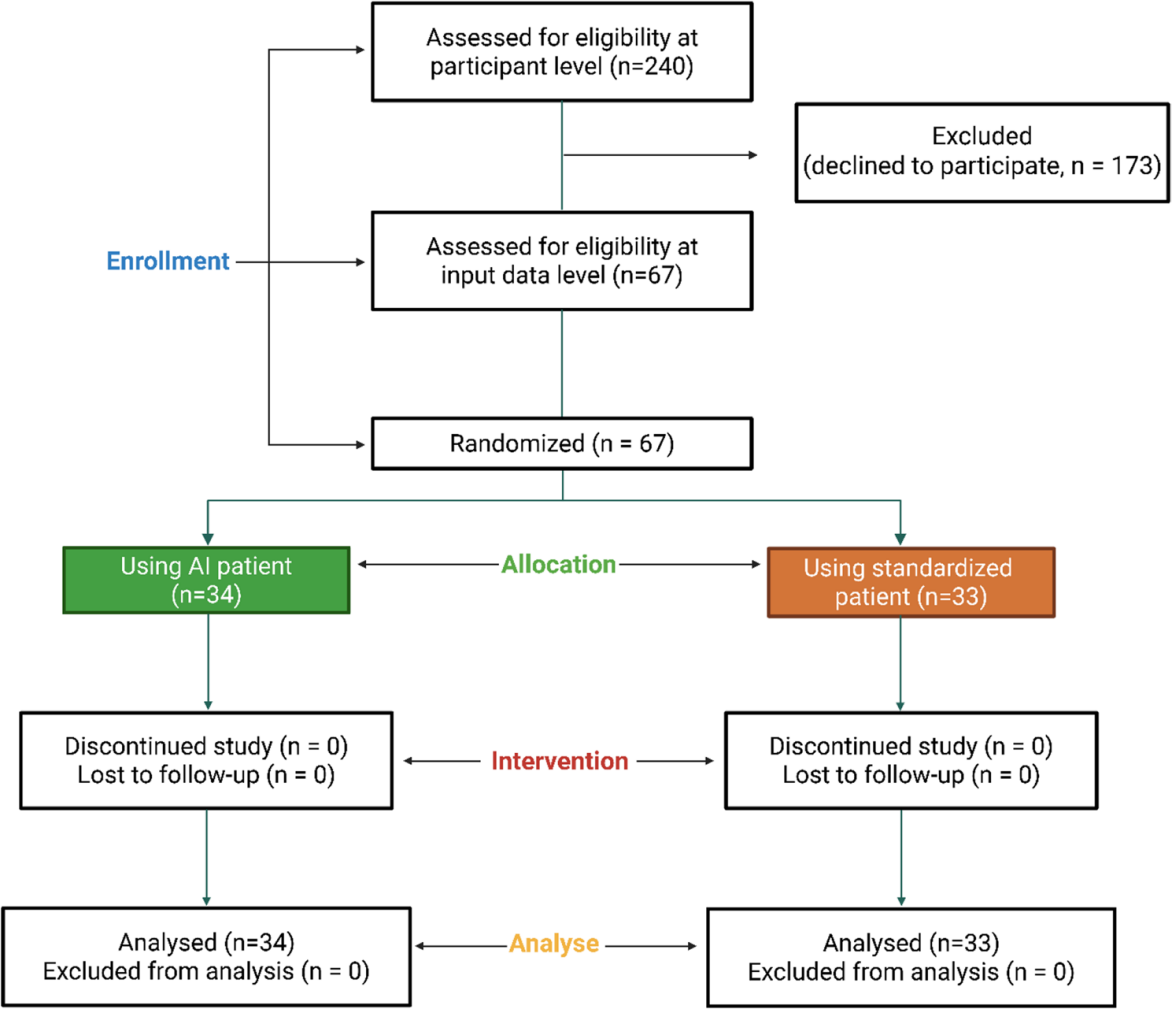
Participant flow diagram

Students were administratively assigned by the university’s Academic Affairs Department into four existing classes, independent of the research process. Following this administrative allocation, randomization was performed at the class level. Each class was randomly assigned to either the intervention group (AI-VSP) or the control group (conventional SP) using a computer-generated random sequence with a 1:1 allocation ratio. The randomization procedure was implemented by an independent researcher who was not involved in participant recruitment, teaching activities, or outcome assessment. The participant enrollment and allocation process are illustrated in Fig. 1.

### Study procedure

The research team collaborated with medical education experts to develop the instructional materials for the study sessions. These included a standardized teaching curriculum, detailed clinical case scenarios, and training protocols for SP based on the designated teaching cases. In addition, assessment checklists were constructed and validated before the study commenced to ensure the consistency and objectivity of student evaluation.

After providing written informed consent, all participants were asked to complete a baseline survey collecting demographic information such as age, gender, and cumulative grade point average (GPA) over the previous two academic years. Students were not informed about the specific purpose of the trial or the evaluation parameters to minimize performance bias. The participating students attended classes according to the same timetable as non-participating students, with scheduling determined independently by the Academic Affairs Department and without involvement from the research team.

Before the teaching intervention, students completed a pre-test designed to assess their baseline knowledge of history-taking. Each teaching session lasted four hours and followed the standardized curriculum approved by the university. During the practical component, students in the intervention group practiced with AI-VSP, while those in the control group worked with conventional SP.

After completing all instructional activities, students took a post-test to evaluate their knowledge improvement following the intervention. Following the session, participants were invited to complete a questionnaire assessing their satisfaction and engagement with the instructional method using a 5-point Likert scale [20].

At the end of the course, all participating students took the OSCE, which included a history-taking station. The OSCE was conducted according to the university’s official examination schedule and procedures. The examiners responsible for assessment were not members of the research team and were blinded to student group allocation. The OSCE results were used as the final performance outcome measure for comparison between the two instructional approaches. Additional details regarding the study procedure and materials are provided in Supplementary Document 1.

### Educational interventions

#### Conventional standardized patient (control group)

The control group received instruction using conventional SP. These individuals were recruited from adults who had previously recovered from the illnesses or from healthy volunteers who expressed interest in participating as SP. All participants underwent formal training and rehearsal sessions to ensure consistent performance. They were compensated for their time and acted as patients for students to practice history-taking. Each SP was thoroughly trained on the assigned clinical scenario, including the presenting symptoms, emotional tone, and expected responses. Before each class, instructors reviewed and verified the accuracy and consistency of each SP’s role to maintain the integrity of the simulation experience.

#### AI-powered virtual standardized patient (intervention group)

The intervention group received instruction using AI-powered virtual SP integrated from the Geeky Medics educational platform. The virtual patient cases were designed, updated, and validated by the research team in collaboration with medical education specialists to ensure alignment with the course objectives. During each teaching session, students interacted with the AI-based virtual patients through preconfigured laptops available in the skills laboratory. The AI-driven system simulated realistic patient dialogues and responses. It allowed students to practice structured history-taking in a dynamic learning environment supported by immediate feedback.

### Outcome measures

The primary outcomes included the knowledge assessment scores obtained from the pre-test and post-test, as well as the end-of-course performance evaluated by the OSCE. Both the pre-test and post-test were designed using multiple-choice questions, each consisting of 10 items with a total possible score ranging from 0 to 10. The questions were developed and validated by medical education experts to ensure appropriate difficulty and content validity for the target learners. The test content was based on the history-taking scenarios taught during the intervention, covering key aspects such as underlying causes, presenting symptoms, and clinical reasoning.

In addition to knowledge improvement, OSCE performance was also analyzed as a primary outcome. The OSCE was conducted at the end of the course following the university’s official examination schedule. Each student completed a history-taking station scored on a 10-point scale, where 0 represented the lowest and 10 the highest performance. The standardized assessment checklist used for scoring was consistent across all examinees. Examiners who graded the OSCE were independent faculty members who were not part of the research team and were blinded to group allocation. The official OSCE results were released after the completion of the examination and were used for the final comparative analysis between groups.

The secondary outcome was student satisfaction and preference toward the instructional method used in their learning session. This was assessed through a structured questionnaire employing a 5-point Likert scale to measure levels of enjoyment, engagement, and perceived usefulness of the AI-VSP or SP experience.

### Statistical analysis

All statistical tests in this study were two-sided, with the significance level set at *p* < 0.05. Statistical analyses were performed via R version 4.4.1 (R Foundation for Statistical Computing, Vienna, Austria) and GraphPad Prism Version 10 (GraphPad Software, Boston, United States). The statistical analysis followed the SAMPL guidelines to ensure clarity and accuracy in reporting [21]. Samples with missing data were excluded from the analysis. Data normality was assessed via the Shapiro–Wilk test. Continuous variables are presented as the mean ± SD. Between-group comparisons were conducted using the Mann–Whitney U test, and within-group comparisons (pre- versus post-intervention) were analyzed using the Wilcoxon signed-rank test. Categorical variables were compared using Fisher’s exact test. To account for potential confounding factors and clustering, generalized linear mixed-effects models were constructed for all primary learning outcomes. Additionally, post hoc analyses were conducted to further assess whether the AI-VSP instructional method achieved learning outcomes that were equivalent or non-inferior to those of traditional SP teaching. The two one-sided tests procedure was applied to compute 95% confidence intervals for the mean differences. This enabled evaluation of equivalence by determining whether the confidence interval fell entirely within the predefined ± 1 point range. Non-inferiority was assessed separately by examining whether the lower confidence limit exceeded the prespecified margin of − 1 point [17]. All results are reported with corresponding 95% confidence intervals.

## Results

A total of 67 medical students participated in the study, including 34 students in the AI-VSP group and 33 students in the conventional SP group. The analysis showed that students in the SP group were significantly older than those in the AI-VSP group. In contrast, there were no statistically significant differences between the two groups in terms of gender distribution, place of residence, or academic performance, including cumulative and semester GPA. Detailed baseline characteristics of the study participants are presented in Table 1.

**Table 1:** Baseline characteristics of study participants

Variables	AI-VSP group (*n* = 34)	SP group (*n* = 33)	*p*-value
Age (years)	20.12 ± 0.84	21.85 ± 4.42	0.018
Female, *n* (%)	16 (47.1)	21 (61.8)	0.222
Family member working in healthcare, *n* (%)	23 (67.6)	24 (70.6)	0.791
Urban residence, *n* (%)	12 (35.3)	18 (52.9)	0.144
Cumulative GPA (previous 2 years)	7.43 ± 0.99	7.52 ± 1.08	0.396
Previous semester GPA	7.40 ± 0.87	7.50 ± 1.03	0.580

The values are presented as the means ± SD or *n* (%) as appropriate

*Abbreviations*: *AI-VSP *artificial intelligence–powered virtual standardized patient, *GPA *grade point average, *SP *standardized patient

**Fig. 2:**
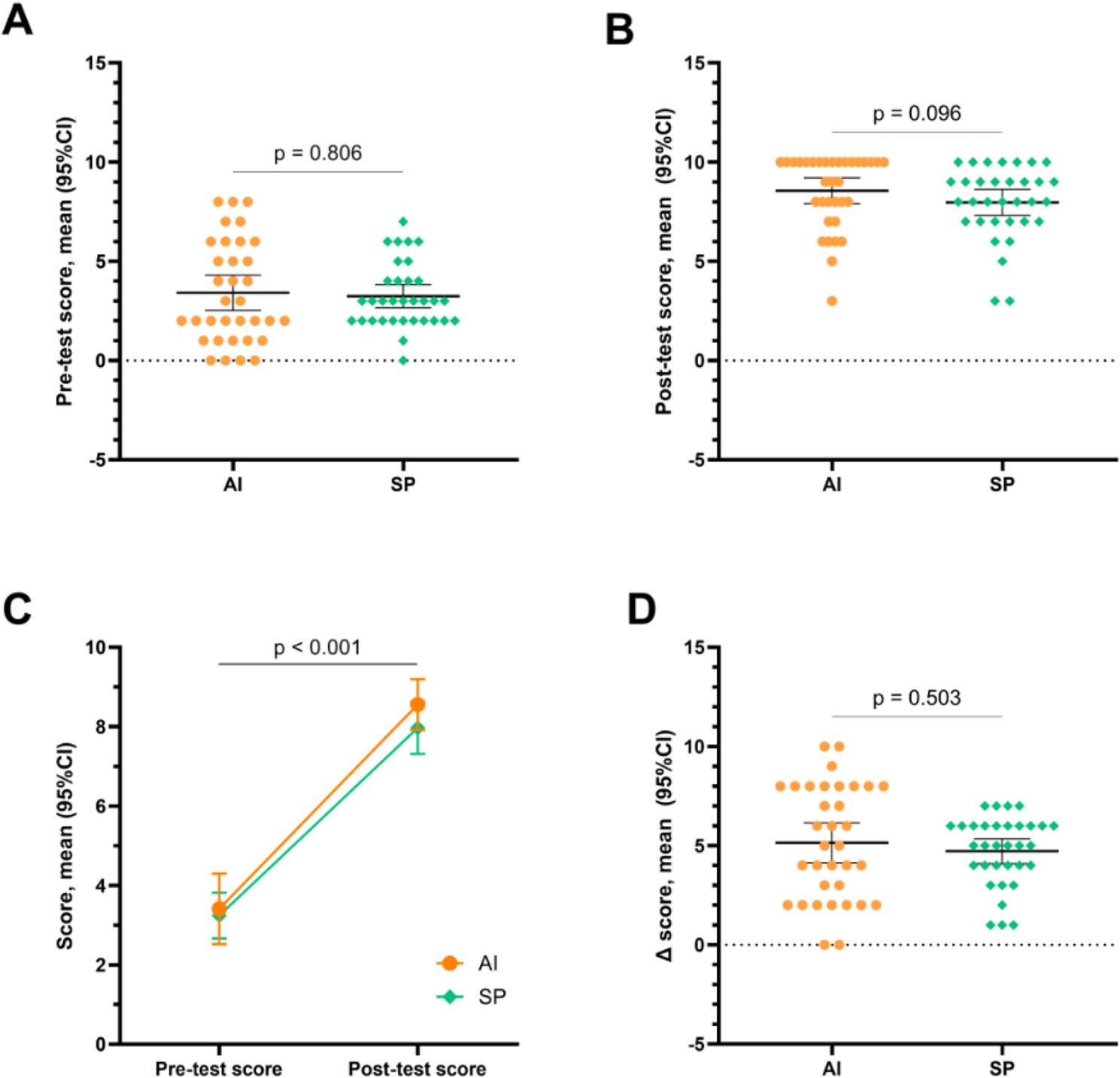
Comparison of learning outcomes between AI-VSP and SP groups. **A** Pre-test scores comparison, (**B**) Post-test scores comparison, (**C**) Pre- and post-test improvement within groups, (**D**) Change in score (∆) between groups

Figure 2A and B illustrate the comparison of student performance between the AI-VSP and SP groups in the pre-test and post-test assessments. The mean pre-test scores were 3.41 ± 2.56 in the AI-VSP group and 3.24 ± 1.64 in the SP group, while the corresponding post-test scores were 8.56 ± 1.85 and 7.97 ± 1.85, respectively. No statistically significant difference was observed between the two groups in either the pre-test or post-test results (*p* > 0.05). As shown in Fig. 2C, both instructional methods resulted in a significant improvement in student performance after the learning sessions, with post-test scores markedly higher than pre-test scores (*p* < 0.001). When comparing the magnitude of score improvement between the two groups, the mean gain was 5.15 ± 2.88 in the AI-VSP group and 4.73 ± 1.75 in the SP group, showing no statistically significant difference (Fig. 2D).

**Fig. 3:**
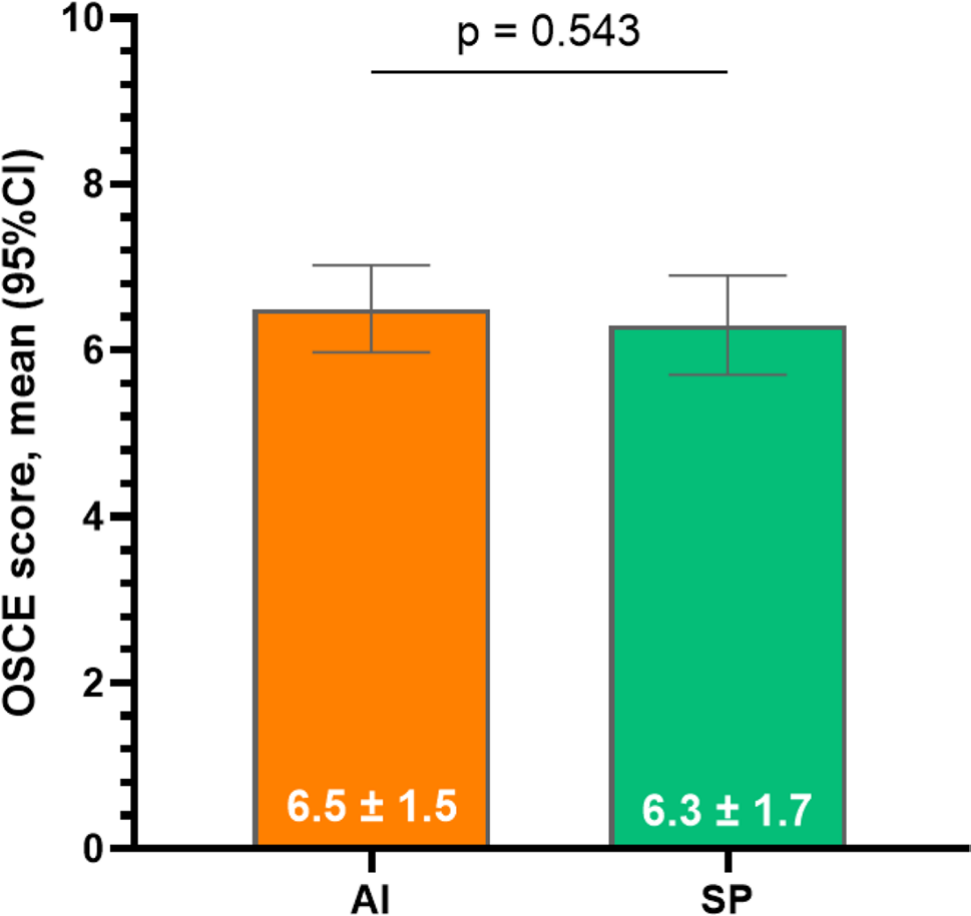
Comparison of OSCE scores between AI-VSP and SP groups

Figure 3 presents the comparison of OSCE performance between the two instructional methods. The mean OSCE scores were 6.5 ± 1.5 in the AI-VSP group and 6.3 ± 1.7 in the SP group. This difference was not statistically significant (*p* = 0.543).

**Fig. 4:**
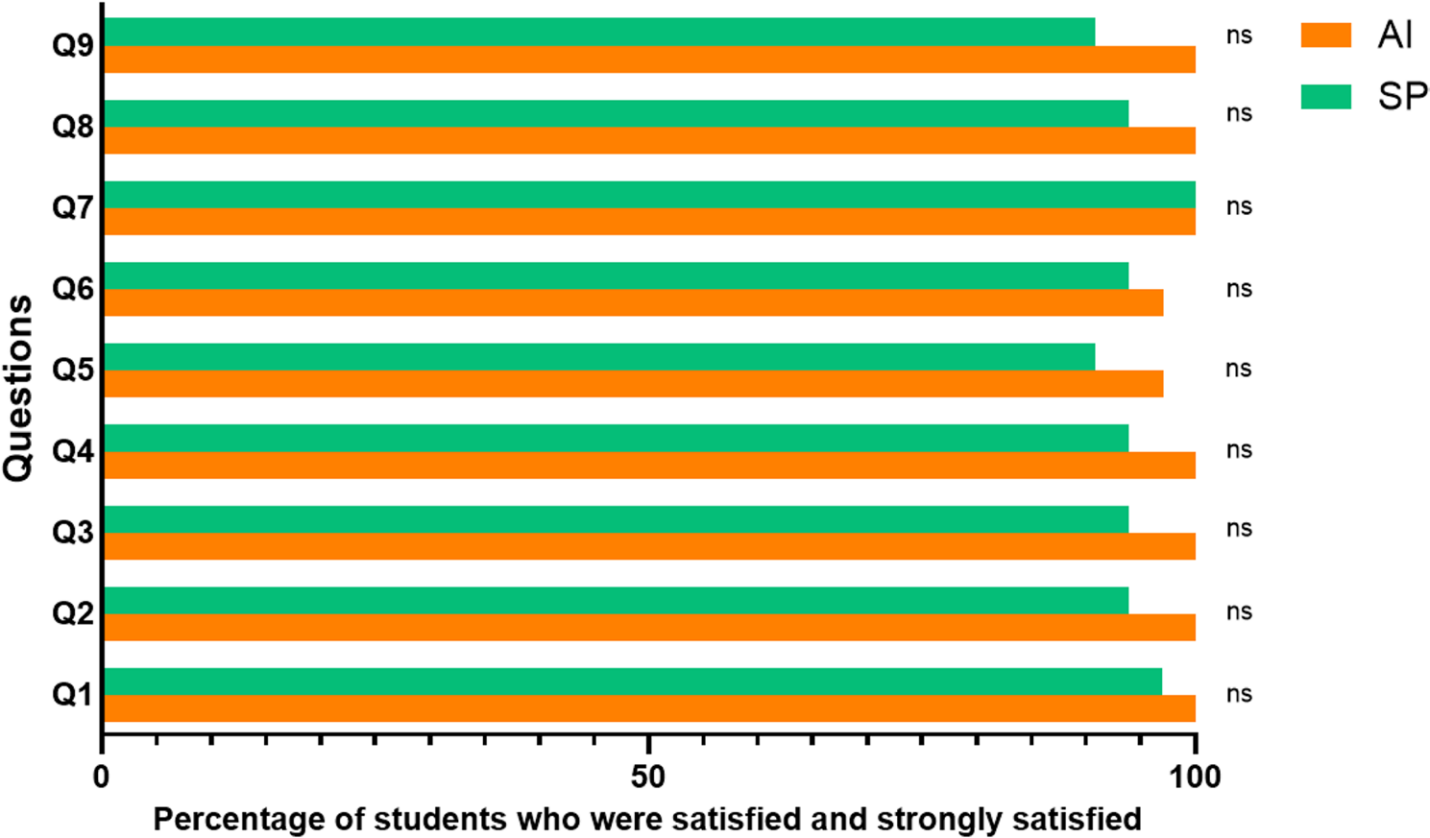
Comparison of student satisfaction between AI-VSP and SP groups

Regarding student satisfaction, the percentage of students who reported being “satisfied” or “very satisfied” with the learning experience did not differ significantly between the AI-VSP and SP groups. Detailed results of the satisfaction survey are summarized in Supplementary Table 1 and visually presented in Fig. 4.

Table 2 shows that the generalized linear mixed-effects models, after adjusting for age and class-level clustering, did not identify any statistically significant differences in learning outcomes between the SP and AI-VSP groups. Specifically, the pre-test (*p* = 0.558), post-test (*p* = 0.078), OSCE score (*p* = 0.219), and Δ score (*p* = 0.421) all showed no meaningful differences between the two groups. Detailed results for the remaining variables are presented in Table 2.

**Table 2:** Generalized linear mixed-effects models for learning outcomes adjusted for class-level clustering

Measure	Parameter	Estimate	SE	z	*p*-value
Pre-test score	Age	0.087	0.085	1.029	0.303
	Groups (SP vs. AI)	-0.320	0.547	-0.585	0.558
Post-test score	Age	0.129	0.071	1.808	0.071
	Groups (SP vs. AI)	-0.812	0.460	-1.765	0.078
OSCE score	Age	0.161	0.060	2.690	0.007
	Groups (SP vs. AI)	-0.475	0.387	-1.230	0.219
Δ score	Age	0.042	0.095	0.440	0.660
	Groups (SP vs. AI)	-0.492	0.611	-0.805	0.421

*Abbreviations*: *AI *artificial intelligence, ∆ difference between post-test and pre-test scores, *OSCE *objective structured clinical examination, *SP *standardized patient

**Fig. 5:**
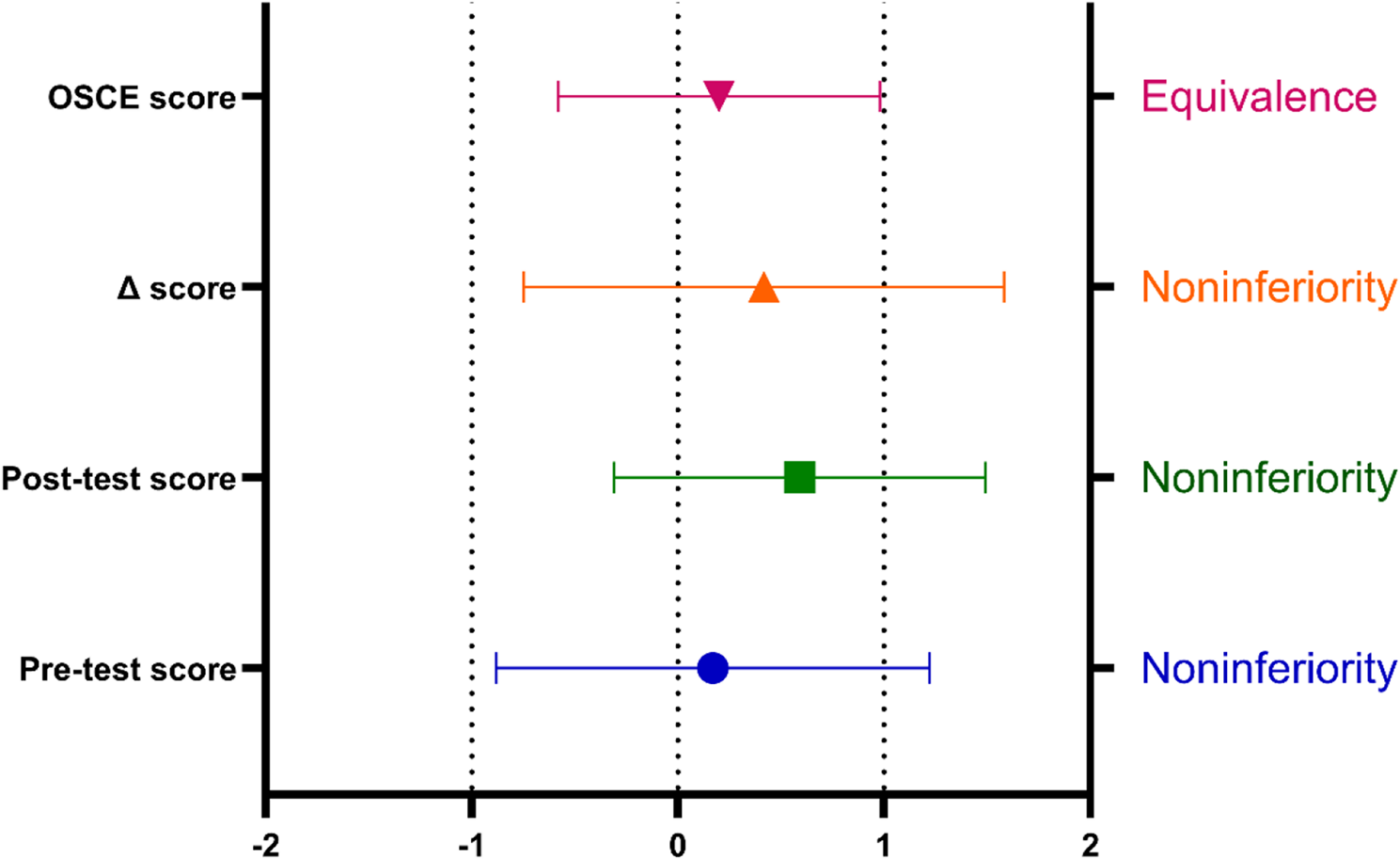
Post hoc equivalence and non-inferiority analysis for learning outcomes between AI-VSP and SP groups

Figure 5 presents an exploratory post hoc analysis indicating that OSCE performance demonstrated equivalence between the AI-VSP and SP groups. For the remaining learning outcomes, AI-VSP showed non-inferiority compared with traditional SP. Similarly, student satisfaction ratings for AI-VSP were non-inferior to those of the SP group. Detailed results are provided in Supplementary Table 2.

## Discussion

This randomized controlled trial is, to our knowledge, the first study conducted in Vietnam to compare the educational effectiveness of AI-VSP with conventional SP in teaching HTS to medical students. Both groups showed similar academic performance at baseline, as reflected by comparable cumulative and semester GPAs, indicating similar academic foundations prior to the intervention.

The results demonstrated no statistically significant difference in pre-test scores between the two groups, supporting the validity of post-intervention comparisons. After the teaching sessions, both groups exhibited significant improvement in knowledge, with post-test scores substantially higher than pre-test scores (*p* < 0.001). Although both instructional approaches led to clear learning gains, the difference in score improvement between the AI-VSP and SP groups was not statistically significant. As shown in Fig. 2C, both methods resulted in marked knowledge enhancement following the intervention, confirming that interactive engagement with either real or virtual SP effectively strengthened students’ HTS. The comparable magnitude of improvement between the two methods (*p* = 0.503) indicates that AI-VSP technology can replicate learning experiences that are not meaningfully different from those achieved with traditional SP instruction. In addition, although there were concerns that the age difference between the two groups and the class-level clustering design might have influenced the intervention effect, the adjusted analysis showed otherwise. After adjustment for age and class-level clustering, the instructional method remained not significantly associated with any primary learning outcome. These findings suggest that the baseline age difference and the clustered study design did not materially affect the overall conclusion that the two instructional approaches had comparable effectiveness.

Previous research has suggested that AI-supported simulations, such as ChatGPT-4o, can be beneficial for practicing clinical communication and SP interactions [22]. ChatGPT-based simulations have been shown to assist learners in developing clinical reasoning, improve communication proficiency, and enhance overall medical training efficiency [23]. The present findings are consistent with these observations and further demonstrate that integrating AI-VSP into medical education is not only feasible but also educationally effective [24].

When evaluating clinical performance using the OSCE, no statistically significant difference was found between the AI-VSP and SP groups (*p* = 0.543). The OSCE, which has been widely used since the 1970s, remains a well-established tool for assessing clinical competence, including history-taking ability [25]. Beyond its role as an assessment instrument, the OSCE is also recognized as an effective learning modality [26]. It has been shown to be well received by students and is considered a reliable method for evaluating end-of-course performance, with broad implementation across medical institutions worldwide [27–29]. At our institution, given the existing educational infrastructure, the OSCE was adopted as the final evaluation method to objectively compare the outcomes of both teaching approaches. The findings reaffirm that both AI-VSP and SP instruction significantly improved students’ learning performance during the course and their end-of-term clinical competence.

Regarding student satisfaction, the percentage of students who reported being satisfied or very satisfied with the learning session did not differ significantly between the AI-VSP and SP groups. This aligns with prior research indicating that students generally perceive AI-based virtual patient systems positively, reporting high levels of engagement and acceptance of this technology [24]. Several studies have also documented favorable learner feedback regarding the use of AI in medical education [30–33]. Due to these promising outcomes, some institutions have begun to develop AI-VSP systems that allow trainees to practice and refine their HTS prior to interacting with real patients [34].

The findings of our study highlight the positive impact of AI integration in teaching HTS. The comparable improvements in knowledge and performance between the AI-VSP and SP groups confirm that AI-enhanced virtual patient platforms can effectively reproduce the interactive and clinically relevant learning experiences of conventional SP encounters. The potential educational applications of this model are substantial. AI can offer flexible and adaptive learning environments that enable students to practice repeatedly without time constraints while receiving immediate feedback based on their communication style and question accuracy. Furthermore, AI systems can personalize the learning process by adjusting the complexity of scenarios according to individual student proficiency, thereby enhancing self-directed learning and learner autonomy.

Despite these encouraging results, the study has several limitations. First, while AI-based instruction is expected to reduce the resource demands associated with SP programs, the present study did not assess cost-effectiveness directly. Previous research has highlighted the potential of AI in healthcare to decrease costs [35], yet its economic impact within medical education remains unclear [36]. Second, the follow-up period was short, allowing only the evaluation of immediate learning outcomes. Consequently, the study cannot determine whether these gains would be sustained over time or lead to improvements in broader educational indices such as GPA, clinical performance with real patients, end-of-course assessments, or future professional competence. Third, assessors were not blinded to the timing of evaluations, which may have introduced measurement bias, although standardized scoring rubrics were used to mitigate this risk. Additionally, because the intervention was delivered within intact student groups, clustering effects cannot be fully excluded and may have contributed to variability in performance, even though statistical analyses were conducted to account for this issue. Finally, as a single-center study conducted in Vietnam, the generalizability of the findings may be limited. Further multicenter trials with larger and more diverse student populations are needed to validate the effectiveness, scalability, and long-term impact of AI-VSP in medical curricula. Nonetheless, this study provides an important foundation and may serve as a catalyst for future research exploring AI-driven simulation in medical training.

## Conclusions

AI-VSP demonstrated educational effectiveness that did not differ significantly from conventional SP in teaching HTS to medical students. Learning outcomes achieved with AI-VSP were not inferior to those obtained with SP-based instruction, and both methods yielded significant post-training improvements in students’ scores. Student satisfaction was also similar between the two approaches, supporting the applicability of AI-VSP in HTS instruction.

## Supplementary Information

Supplementary Material 1.

Supplementary Material 2.

Supplementary Material 3.

## Data Availability

The datasets used and/or analyzed during the current study are available from the corresponding author or first author (hai.dang@utas.edu.vn or dangnngochai@dtu.edu.vn or ngochai123dc@gmail.com) upon reasonable request.

## Abbreviations

AI: Artificial intelligence
AI-VSP: Artificial Intelligence–Powered Virtual Standardized Patient
CONSORT: Consolidated standards of reporting
GPA: Grade point average
HTS: History-taking skills
OSCE: Objective structured clinical examination
SD: Standard deviation
SP: Standardized patient
