# Supplementary material for: Artificial intelligence–powered virtual standardized patients in teaching history-taking skills to medical students: a randomized controlled trial
Source: BMC Med Educ. 2026 Apr 30;26:984. doi: 10.1186/s12909-026-09305-5 (PMC13274040; doi:10.1186/s12909-026-09305-5)
Supplement: Supplementary file 1 — Supplementary Material 1. [file 12909_2026_9305_MOESM1_ESM.docx]

**Supplementary Table 1. Student satisfaction and preference after the learning session**

| **Questions** | **AI-powered virtual standardized patient** | | **Conventional standardized patient** | | **P value** |
| --- | --- | --- | --- | --- | --- |
|  | **Agree/Strongly agree** | **Neutral/Disagree/Strongly disagree** | **Agree/Strongly agree** | **Neutral/Disagree/Strongly disagree** |  |
| Q1 | 34 (100) | 0 (0) | 32 (97.0) | 1 (3.0) | 0.493 |
| Q2 | 34 (100) | 0 (0) | 31 (93.9) | 2 (6.1) | 0.239 |
| Q3 | 34 (100) | 0 (0) | 31 (93.9) | 2 (6.1) | 0.239 |
| Q4 | 34 (100) | 0 (0) | 31 (93.9) | 2 (6.1) | 0.239 |
| Q5 | 33 (97.1) | 1 (2.9) | 30 (90.9) | 3 (9.1) | 0.356 |
| Q6 | 33 (97.1) | 1 (2.9) | 31 (93.9) | 2 (6.1) | 0.614 |
| Q7 | 34 (100) | 0 (0) | 33 (100) | 0 (0) | >0.99 |
| Q8 | 34 (100) | 0 (0) | 31 (93.9) | 2 (6.1) | 0.239 |
| Q9 | 34 (100) | 0 (0) | 30 (90.9) | 3 (9.1) | 0.114 |

Q1. I felt interested in this learning method; Q2. I would like to continue learning with this method in future sessions; Q3. This learning method made the class more engaging and accessible; Q4. The method helped me understand the history-taking process more clearly; Q5. I felt more confident in taking a patient history after the session; Q6. I could immediately apply the knowledge gained to simulated clinical situations; Q7. I was satisfied with the content and organization of the session; Q8. I considered this session to be effective and worth my time; Q9. Overall, I was satisfied with the learning experience in history-taking.
