# Supplementary material for: Artificial intelligence–powered virtual standardized patients in teaching history-taking skills to medical students: a randomized controlled trial
Source: BMC Med Educ. 2026 Apr 30;26:984. doi: 10.1186/s12909-026-09305-5 (PMC13274040; doi:10.1186/s12909-026-09305-5)
Supplement: Supplementary file 2 — Supplementary Material 2. [file 12909_2026_9305_MOESM2_ESM.docx]

**Supplementary Table 2. Post hoc non-inferiority and equivalence analyses for satisfaction items**

| **Question** | **AI (%)** | **SP (%)** | **Diff (AI–SP) (%)** | **95% CI (%)** | **p-value** | **Conclusion** |
| --- | --- | --- | --- | --- | --- | --- |
| Q1 | 100 | 97.0 | 3.0 | –2.8 – 8.9 | 0.306 | Equivalent |
| Q2 | 100 | 93.9 | 6.1 | –2.1 - 14.2 | 0.145 | Non-inferior |
| Q3 | 100 | 93.9 | 6.1 | –2.1 - 14.2 | 0.145 | Non-inferior |
| Q4 | 100 | 93.9 | 6.1 | –2.1 - 14.2 | 0.145 | Non-inferior |
| Q5 | 97.1 | 90.9 | 6.1 | –5.2 - 17.5 | 0.288 | Non-inferior |
| Q6 | 97.1 | 93.9 | 3.1 | –6.8 - 13.0 | 0.537 | Non-inferior |
| Q7 | 100 | 100 | 0.0 | 0.0 - 0.0 | NaN | Equivalent |
| Q8 | 100 | 93.9 | 6.1 | –2.1 - 14.2 | 0.145 | Non-inferior |
| Q9 | 100 | 90.9 | 9.1 | –0.7 - 18.9 | 0.072 | Non-inferior |

Q1. I felt interested in this learning method; Q2. I would like to continue learning with this method in future sessions; Q3. This learning method made the class more engaging and accessible; Q4. The method helped me understand the history-taking process more clearly; Q5. I felt more confident in taking a patient history after the session; Q6. I could immediately apply the knowledge gained to simulated clinical situations; Q7. I was satisfied with the content and organization of the session; Q8. I considered this session to be effective and worth my time; Q9. Overall, I was satisfied with the learning experience in history-taking.
