# Supplementary material for: Artificial intelligence–powered virtual standardized patients in teaching history-taking skills to medical students: a randomized controlled trial
Source: BMC Med Educ. 2026 Apr 30;26:984. doi: 10.1186/s12909-026-09305-5 (PMC13274040; doi:10.1186/s12909-026-09305-5)
Supplement: Supplementary file 3 — Supplementary Material 3. [file 12909_2026_9305_MOESM3_ESM.docx]

**PRE-TEACHING ASSESSMENT (PRE-TEST)**

**Question 1:** In acute pancreatitis, the pain typically radiates to which of the following regions?

1. **Left shoulder**
2. **Flank region**
3. **Back**
4. **Lower abdomen**
5. **Unknown**

**Question 2:** The characteristic pattern of pain in acute pancreatitis is:

1. **Intermittent pain**
2. **Pain that subsides and then recurs**
3. **Continuous pain**
4. **Pain that increases and decreases cyclically in intensity**
5. **Unknown**

**Question 3:** The intensity of pain in acute pancreatitis is typically described as:

1. **Mild**
2. **Dull and tolerable**
3. **Moderate to severe**
4. **Almost painless**
5. **Unknown**

**Question 4:** The most common pain-relieving posture in patients with acute pancreatitis is:

1. **Lying supine with hands on the abdomen**
2. **Sitting and leaning forward**
3. **Restless, with no pain-relieving posture**
4. **Standing upright**
5. **Unknown**

**Question 5:** The usual onset circumstances of pain in acute pancreatitis are:

1. **Pain after an argument with a lover**
2. **Sudden onset**
3. **Occurs after waking up in the morning**
4. **Pain during urination**
5. **Unknown**

**Question 6:** Which of the following gastrointestinal symptoms commonly accompanies acute pancreatitis?

1. **Intermittent diarrhea**
2. **Nausea and vomiting**
3. **Painful urination**
4. **Missed menstrual period**
5. **Unknown**

**Question 7:** Which of the following is NOT a common manifestation of acute pancreatitis?

1. **Epigastric pain**
2. **High fever and chills**
3. **Jaundice with pale stools**
4. **Pain relieved by lying supine**
5. **Unknown**

**Question 8:** Which of the following characteristics is consistent with acute pancreatitis**?**

1. **Pain associated with nausea**
2. **Patient cannot remain still due to pain**
3. **Pain radiating toward the genital area with painful urination**
4. **Sudden severe pain accompanied by jaundice**
5. **Unknown**

**Question 9:** Which of the following factors should be explored when assessing the characteristics of pain in acute pancreatitis?

1. **Location, radiation, intensity, and onset time**
2. **Patient’s favorite food**
3. **History of food allergies**
4. **Recent stress level**
5. **Unknown**

**Question 10:** Which of the following questions is appropriate for assessing the severity of epigastric pain?

1. **Do you feel nauseated?**
2. **How would you rate your pain on a scale from 1 to 10?**
3. **Have you ever experienced similar pain before?**
4. **Do you have jaundice?**
5. **Unknown**

**POST-TEACHING ASSESSMENT (POST-TEST)**

**Question 1:** In acute pancreatitis, the pain typically radiates to which of the following regions?

**A. Left shoulder**

**B. Flank region**

**C. Back**

**D. Lower abdomen**

**Question 2:** The characteristic pattern of pain in acute pancreatitis is:

**A. Intermittent pain**

**B. Pain that subsides and then recurs**

**C. Continuous pain**

**D. Pain that increases and decreases cyclically in intensity**

**Question 3:** The intensity of pain in acute pancreatitis is typically described as:

**A. Mild**

**B. Dull and tolerable**

**C. Moderate to severe**

**D. Almost painless**

**Question 4:** The most common pain-relieving posture in patients with acute pancreatitis is:

**A. Lying supine with hands on the abdomen**

**B. Sitting and leaning forward**

**C. Restless, with no pain-relieving posture**

**D. Standing upright**

**Question 5:** The usual onset circumstances of pain in acute pancreatitis are:

**A. Pain after an argument with a lover**

**B. Sudden onset**

**C. Occurs after waking up in the morning**

**D. Pain during urination**

**Question 6:** Which of the following gastrointestinal symptoms commonly accompanies acute pancreatitis?

**A. Intermittent diarrhea**

**B. Nausea and vomiting**

**C. Painful urination**

**D. Missed menstrual period**

**Question 7:** Which of the following is NOT a common manifestation of acute pancreatitis?

**A. Epigastric pain**

**B. High fever and chills**

**C. Jaundice with pale stools**

**D. Pain relieved by lying supine**

**Question 8:** Which of the following characteristics is consistent with acute pancreatitis?

**A. Pain associated with nausea**

**B. Patient cannot remain still due to pain**

**C. Pain radiating toward the genital area with painful urination**

**D. Sudden severe pain accompanied by jaundice**

**Question 9:** Which of the following factors should be explored when assessing the characteristics of pain in acute pancreatitis?

**A. Location, radiation, intensity, and onset time**

**B. Patient’s favorite food**

**C. History of food allergies**

**D. Recent stress level**

**Question 10:** Which of the following questions is appropriate for assessing the severity of epigastric pain?

**A. Do you feel nauseated?**

**B. How would you rate your pain on a scale from 1 to 10?**

**C. Have you ever experienced similar pain before?**

**D. Do you have jaundice?**

**CASE SUMMARY**

A 54-year-old retired male, formerly a teacher, presented to the Emergency Department with severe epigastric pain.

The pain began suddenly about 90 minutes before admission, described as a sharp, stabbing, cramping sensation located in the epigastric region. The patient reported that the pain radiated to the back, was continuous and progressively worsening, and was exacerbated by deep inspiration or movement, but partially relieved when leaning forward. He rated the intensity as 11 out of 10. This was his first episode of such pain.

Associated symptoms included severe nausea and a sensation of fever with sweating, but no vomiting. He also reported shortness of breath even at rest, though bowel movements were normal earlier that morning. Over the past four weeks, he had intentionally lost 3 kg through exercise.

He denied jaundice, pruritus, dysphagia, reflux, changes in bowel habits, gastrointestinal bleeding, dysuria, confusion, or recent travel.

His past medical history included gallstones several months prior (though the current pain was much more severe) and type 2 diabetes mellitus diagnosed five years ago. He takes Metformin 1000 mg twice daily (8 a.m. and 4 p.m.) and Dapagliflozin 10 mg daily (8 a.m.), attends monthly follow-ups for medication refills, and has no known drug allergies.

Family history was unremarkable.

Socially, he lives with his wife and two children (a 17-year-old son and a 21-year-old daughter). He does not smoke, drinks a few cans of beer occasionally during celebrations, has recently started an exercise program for weight loss, and does not use recreational drugs.

**OSCE EVALUATION CHECKLIST**

| **NO.** | **CHECKLIST ITEM** | **COMPLETED** | **NOT COMPLETED** |
| --- | --- | --- | --- |
| **A. OPENING – COMMUNICATION** | | | |
| 1 | Greeting and self-introduction | ☐ | ☐ |
| 2 | Explaining the purpose of the encounter | ☐ | ☐ |
| 3 | Asking for the patient’s permission before starting | ☐ | ☐ |
| **B. EXPLORING THE MAIN SYMPTOM (ABDOMINAL PAIN)** | | | |
| 4 | Asking about the site of pain (epigastric area) | ☐ | ☐ |
| 5 | Asking about the onset of pain (sudden onset, 90 minutes ago) | ☐ | ☐ |
| 6 | Asking about the character of pain (sharp, cramping) | ☐ | ☐ |
| 7 | Asking about the severity of pain (11/10) | ☐ | ☐ |
| 8 | Asking about radiation of pain (to the back) | ☐ | ☐ |
| 9 | Asking about aggravating/relieving factors (worse on deep inspiration, relieved by leaning forward) | ☐ | ☐ |
| 10 | Asking about the progression of pain (continuous, worsening) | ☐ | ☐ |
| **C. ASSOCIATED / RELATED SYMPTOMS** | | | |
| 11 | Asking about nausea | ☐ | ☐ |
| 12 | Asking about fever or chills | ☐ | ☐ |
| 13 | Asking about shortness of breath | ☐ | ☐ |
| 14 | Asking about the most recent bowel movement | ☐ | ☐ |
| **D. PAST MEDICAL HISTORY AND MEDICATIONS** | | | |
| 15 | Asking about personal medical history | ☐ | ☐ |
| 16 | Asking about family medical history | ☐ | ☐ |
| **E. IDEAS, CONCERNS, EXPECTATIONS** | | | |
| 17 | Asking what the patient thinks is happening (Ideas) | ☐ | ☐ |
| 18 | Asking what the patient is worried about (Concerns) | ☐ | ☐ |
| 19 | Asking what the patient expects from the consultation (Expectations) | ☐ | ☐ |
| **F. ATTITUDE – COMMUNICATION SKILLS** | | | |
| 20 | Communicates gently, does not interrupt the patient | ☐ | ☐ |
| 21 | Uses clear, respectful language | ☐ | ☐ |

Each completed item is awarded 1 point, giving a total score of 21 points. This total will then be converted to a 10-point scale. For example: 17/21 = 8.1, rounded to the nearest tenth.
